# Comment on Onisor et al. Effectiveness and Clinical Performance of Erythritol Air-Polishing in Non-Surgical Periodontal Therapy: A Systematic Review of Randomized Clinical Trials. *Medicina* 2022, *58*, 866

**DOI:** 10.3390/medicina61091534

**Published:** 2025-08-27

**Authors:** Jamal M. Stein

**Affiliations:** 1 Praxiszentrum für Implantologie, Parodontologie & Prothetik, Schumacherstraße 14, 52062 Aachen, Germany; jstein@ukaachen.de; 2 Department of Operative Dentistry, Periodontology and Preventive Dentistry, University Hopsital RWTH Aachen, Pauwelsstraße 30, 52074 Aachen, Germany

Unfortunately, an article by Onisor et al. 2022 [1] published in *Medicina* includes a severe problem by evaluating the data of different studies including a randomized controlled clinical trial by our group [2] in an inappropriate way.

In Figure 3 the authors compare the CAL, PD and BOP values 6 months after therapy between the control group (subgingival instrumentation only) and the test group (subgingival instrumentation + erythritol powder air-polishing). In the Discussion (in the first sentence in the Discussion Section), these results are interpreted as indicating the “efficiency” of therapy. However, the efficiency of therapy can only be estimated by the changes in these parameters from baseline to follow-up values (unless the baseline values are the same, which is—of course—not the case). Thus, the comparison of the changes in these parameters would be appropriate, but no data on this issue can be found in the article.

For example, the meta-analysis implies that in terms of CAL, our study [2] favors the control group (4.01 mm) and not the test group (4.26). However, the change in CAL from baseline to follow-up was 0.54 mm in the control group and 0.77 mm in the test group, meaning that the efficiency of the therapy favors the test group (not the control group), irrespective of any statistical significance.

To summarize this issue, comparing the follow-up values alone cannot give conclusions on “efficiency” (Discussion) or “effectiveness” (title). This could only be carried out if baseline values were the same in all studies included in the meta-analysis. However, the authors do not report any baseline-matched analyses or change scores.

Therefore, unfortunately, this seems to be a major problem in the paper which leads to conclusions that are not justified. Thus, the presentation of the data of our and the other studies included in the meta-analysis will lead to mis-interpretation.
